# Acid rain and air pollution: 50 years of progress in environmental science and policy

**DOI:** 10.1007/s13280-019-01244-4

**Published:** 2019-09-21

**Authors:** Peringe Grennfelt, Anna Engleryd, Martin Forsius, Øystein Hov, Henning Rodhe, Ellis Cowling

**Affiliations:** 1 IVL Swedish Environmental Research Institute, PO Box 53021, 40014 Gothenburg, Sweden; 2 Swedish Environmental Protection Agency, Virkesvägen 2F, 10648 Stockholm, Sweden; 3 Finnish Environment Institute, Latokartanonkaari 11, 00790 Helsinki, Finland; 4 The Norwegian Meteorological Institute, P.O. Box 43, Blindern, 0313 Oslo, Norway; 5 Department of Meteorology, Stockholm University, 10691 Stockholm, Sweden; 6 Department of Forestry and Environmental Resources, NC State University, 5211 Glenhope Court, Cary, NC 27511 USA

**Keywords:** Acid rain, Air pollution, Critical loads, Ecosystems, Integrated assessment modelling, Monitoring, Nitrogen, Policy development, Sulphur

## Abstract

Because of its serious large-scale effects on ecosystems and its transboundary nature, acid rain received for a few decades at the end of the last century wide scientific and public interest, leading to coordinated policy actions in Europe and North America. Through these actions, in particular those under the UNECE Convention on Long-range Transboundary Air Pollution, air emissions were substantially reduced, and ecosystem impacts decreased. Widespread scientific research, long-term monitoring, and integrated assessment modelling formed the basis for the policy agreements. In this paper, which is based on an international symposium organised to commemorate 50 years of successful integration of air pollution research and policy, we briefly describe the scientific findings that provided the foundation for the policy development. We also discuss important characteristics of the science–policy interactions, such as the critical loads concept and the large-scale ecosystem field studies. Finally, acid rain and air pollution are set in the context of future societal developments and needs, e.g. the UN’s Sustainable Development Goals. We also highlight the need to maintain and develop supporting scientific infrastructures.

## Introduction

Acid rain was one of the most important environmental issues during the last decades of the twentieth century. It became a game changer both scientifically and policy-wise. For some time, particularly during the 1980s, acid rain was by many considered to be one of the largest environmental threats of the time. Observations of fish extinction in Scandinavian surface waters and forest dieback on the European Continent were top stories in the news media. Even in North America acid rain received large public and policy attention.

**Fig. 1:**
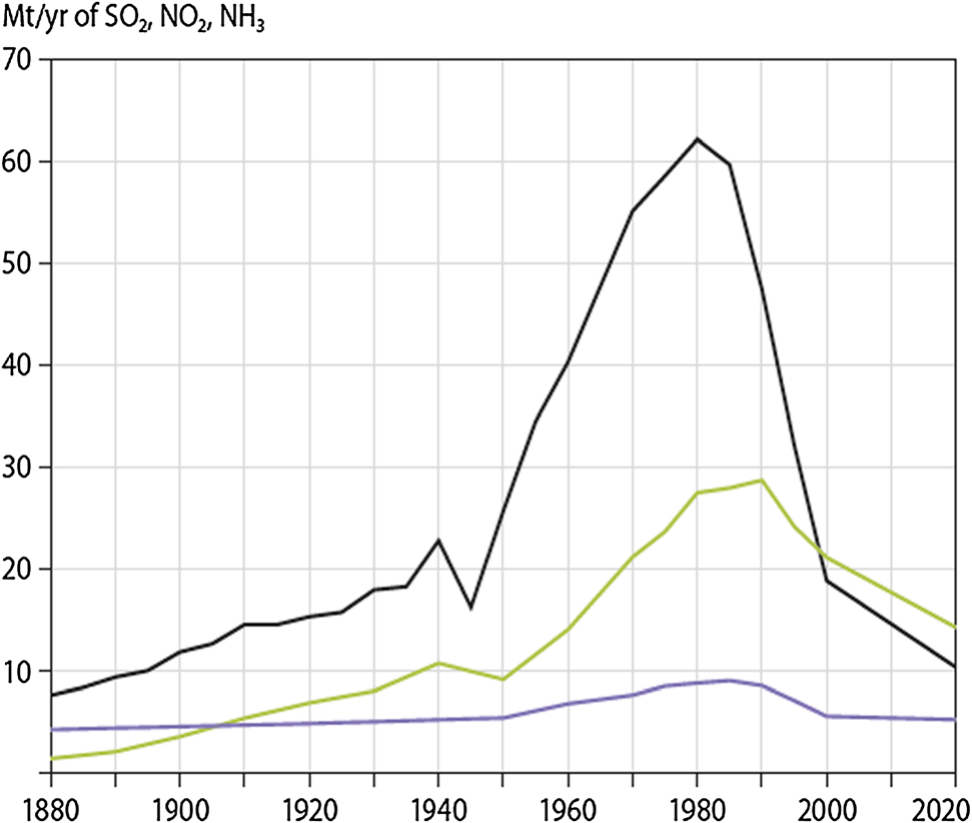
European emissions of sulphur dioxide (SO_2_—black), nitrogen oxides (NO_x_, calculated as NO_2_—green) and ammonia (NH_3_—blue) 1880–2020 (updated from Fig. 2 in Schöpp et al. 2003)

During the cold war, with almost no contacts between East and West, acid rain broke the ice and formed an opening for scientific and political collaboration, resulting in a treaty under the United Nations’ Economic Commission for Europe (UNECE), the Convention on Long-range Transboundary Air Pollution (often mentioned as CLRTAP but in this paper we call it the Air Convention) signed in 1979. Eight protocols have been signed under the Air Convention committing parties to take far-reaching actions, not only with respect to acid rain but also with respect to several other air pollution problems (Table 1). Emissions of all key air pollutants have been reduced significantly and for the most important acidifying compound, sulphur dioxide, emissions in Europe have decreased by 80% or more since the peaks around 1980–1990 (Fig. 1).

**Table 1:** The Convention on Long-Range Transboundary Air Pollution and Its Protocols

Agreement	Content	Comment
The 1979 convention on long-range transboundary air pollution	Frame convention	51 parties to the convention
The 1999 Gothenburg protocol to abate acidification, eutrophication, and ground-level ozone	Emissions of SO_2_, NO_x_, NH_3_, and volatile organic compounds (VOC), Amendment also fine particulates	Amended in 2012
The 1998 Aarhus protocol on persistent organic pollutants (POPs)	A selected number of persistent organic compounds	Amended in 2009
The 1998 Aarhus protocol on heavy metals	Control of a selected number of heavy metals	Amended in 2012
The 1994 Oslo protocol on further reduction of sulphur emissions	The protocol sets ceilings for SO_2_ emissions based on CL and IAM	
The 1991 Geneva protocol concerning the control of emissions of volatile organic compounds or their transboundary fluxes	30% reduction in VOC emissions	
The 1988 Sofia protocol concerning the control of emissions of nitrogen oxides or their transboundary fluxes	Stipulates no further increase in NO_x_ emissions	
The 1985 Helsinki protocol on the reduction of sulphur emissions or their transboundary fluxes by at least 30%	SO_2_ control by 30% between 1980 and 1993	
The 1984 Geneva protocol on long-term financing of the cooperative programme for monitoring and evaluation of the long-range transmission of air pollutants in Europe (EMEP)	Stipulates financial support to the EMEP centres

**Fig. 2:**
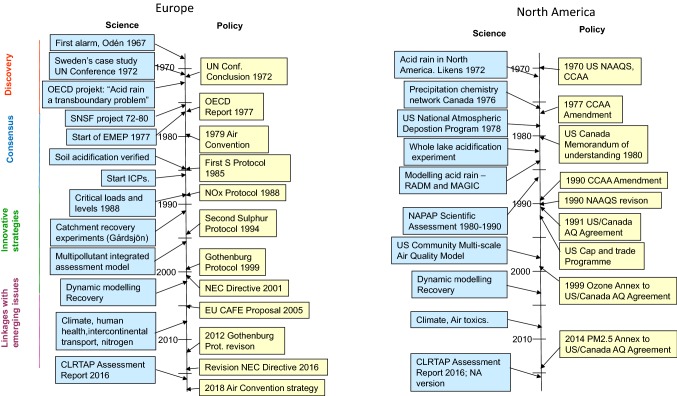
The timeline of science and policy interactions in Europe and North America 1967–2018. (updated from Driscoll et al. 2012). Abbreviations not occurring in text. NAAQS: National Ambient Air Quality Standards under the US Clean Air Act; CCAA: Canadian Clean Air Act; RADM: Regional Atmospheric Deposition Model; MAGIC Model of Acidification of Groundwater in Catchments. It should be mentioned that Canada and US are both parties to the Air Convention and they have also signed and ratified most of its protocols

In this paper, we present and discuss how the acid rain problem became a key environmental issue among industrial countries from the late 1960s and the following decades (Fig. 2). We view the problem from a science-to-policy interaction perspective, based on a Symposium in Stockholm in the autumn 2017 organised to manifest 50 years of international air pollution science and policy development. The Symposium involved both a testimony from a number of those involved in science and policy during the first decades of the history but also a discussion of what we have learned and how the experience can be used in the future. Further information about the symposium and its outcome can be found at http://acidrain50years.ivl.se.

Our historical review will be limited to some of the issues brought up at the Symposium. For more information on the early history see Cowling (1982). A comprehensive description of the acid rain history has recently been published by Rothschild (2018). The history of the first 30 years of the science–policy interactions under the Air Convention is also described in Sliggers and Kakebeeke (2004).

## Short historical review

### The discovery and the early acid rain history

**Fig. 3:**
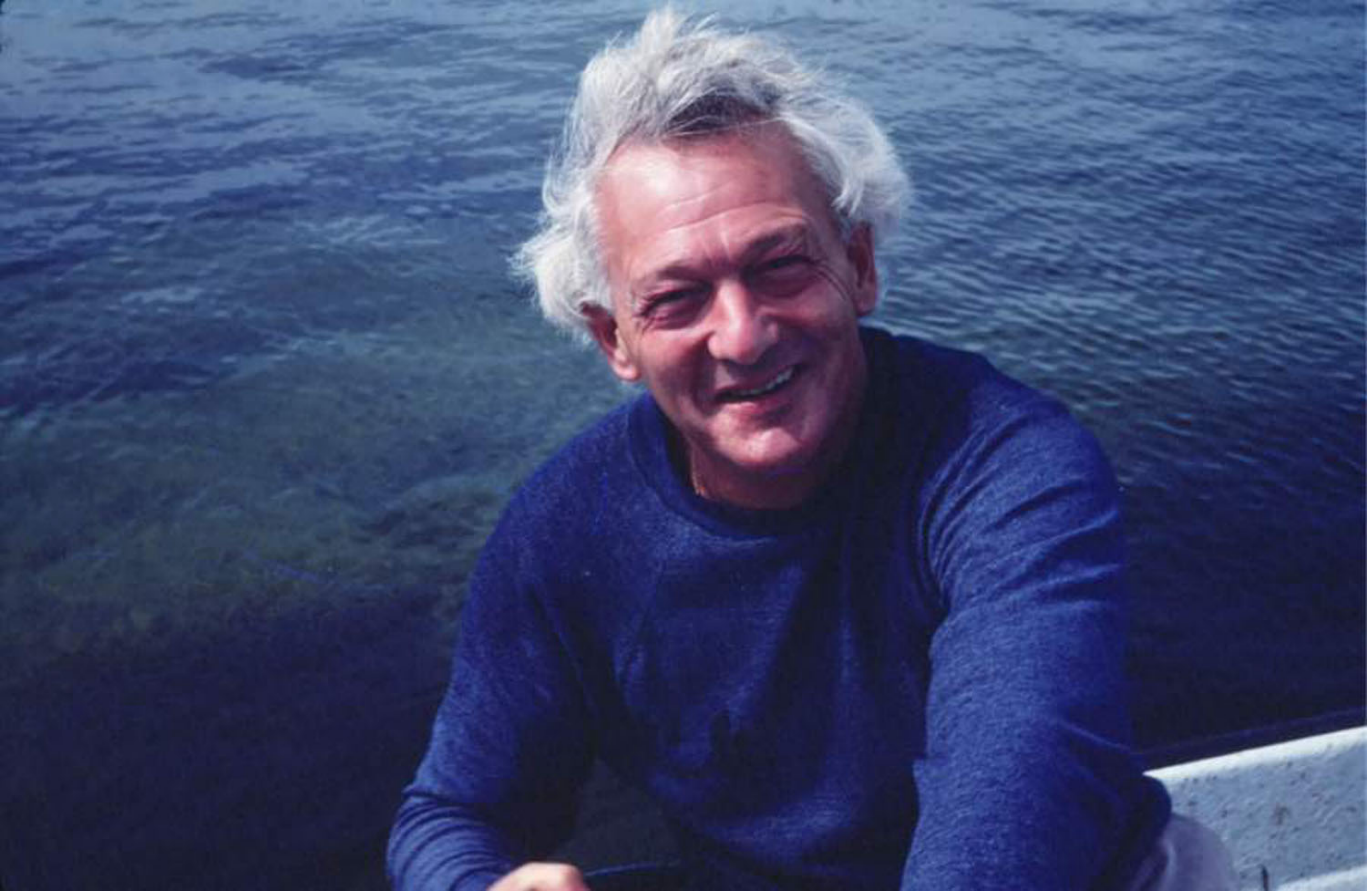
Svante Odén around 1970 (photo Ellis B. Cowling)

In a deliberatively provocative article in the Swedish newspaper Dagens Nyheter in October 1967, entitled “An Insidious Chemical Warfare Among the Nations of Europe”, the Swedish scientist Svante Odén (Fig. 3) described a new and threatening environmental problem—Acid Rain. He pointed to the significant decrease in pH of rainwater and surface waters that had occurred over the previous decade and linked it to the large and increasing emissions of sulphur dioxide in Europe.

The discovery received immediate attention by the Swedish government and, a few weeks after Odén’s article, the minister of industry presented the issue at the Organisation for Economic Cooperation and Development (OECD), but it did not receive any political attention at that time. The issue was also brought up in OECD’s Air Pollution Management Committee by the Swedish delegate Göran Persson. Also, here the message was met by scepticism and the common opinion among the members in the committee was that sulphur dioxide was a local problem, which easily could be solved by tall stacks. It was not until Persson felt he was going to “loose the case” he “played his last card” and pointed to the observations of intercontinental transport of radioactivity from the Chinese nuclear bomb experiments. The opinion then changed and the meeting agreed that acid rain might be an issue to look into. From now on, OECD and the western world realised that air pollution might be a problem of international political dimensions.

Odén’s discoveries were to a large extent based on the regional precipitation networks that were running in Sweden and Europe. In 1947, the Swedish scientist Hans Egnér set up a Swedish network to investigate the importance of atmospheric deposition for the fertilisation of crops. In 1954, the network was expanded forming the European Air Chemistry Network (EACN) through initiatives by Egnér, Carl Gustav Rossby, and Erik Eriksson (Egnér and Eriksson 1955; see also Engardt et al. 2017). Data from these networks together with a Scandinavian surface water network set up by Odén in 1961 formed the basis for Odén’s observations on the ongoing acidification (Odén 1968).

Acid rain and many of its ecological effects were, however, recognised long before 1967–1968. In fact, many features of the acid rain phenomenon were first discovered by an English chemist, Robert Angus Smith, in the middle of the nineteenth century! In 1852, Smith published a detailed report on the chemistry of rain in and around the city of Manchester, England. Twenty years later, in a very detailed book titled “Air and Rain: The Beginnings of a Chemical Climatology”, Smith first used the term “acid rain” and enunciated many of the principal ideas that are part of our present understanding of this phenomenon (Smith 1872). Unfortunately, however, Smith’s pioneering book was substantially ignored by nearly every subsequent investigator.

In Norway salmon catches decreased substantially in the early 1900s and in 1927, Professor Knut Dahl hypothesised that acidification of surface waters could be a factor of importance for the extinction of fish. Later Alf Dannevig assumed that “The acidity of a lake is dependent on the acidity of the rainwater and the contributions from the soil” (Dannevig 1959).

Based on detailed field observations and experimental studies both in England and in Canada, beginning in 1955 and continuing through 1963, Eville Gorham and his colleagues built a significant foundation for contemporary understanding of the causes of acid precipitation and its impacts on aquatic ecosystems, agricultural crops, soils, and even human health (Gorham 1981; Cowling 1982). Thus, Gorham and his colleagues as well as Dahl and Dannevig had discovered major aspects of the causes of contemporary changes in the chemistry of atmospheric emissions and deposition and their effects on aquatic ecosystems.

But these pioneering contributions, like those of Smith a century earlier, were not generally recognised—neither by scientists nor by society in general. Gorham’s researches, like those of Smith a century before, were met by what Gorham himself acknowledged as a “thundering silence”, not only by the scientific community, but also by the public at large.

It was not until 1967 and 1968 when Svante Oden published both his deliberatively provocative article in Dagens Nyheter and his carefully documented Ecological Committee Report (Odén 1968) that the acid rain problem was brought to both public and scientific considerations. The report included a huge body of scientific and policy-relevant evidence that long-distance transport and deposition of acidifying pollutants were causing significant environmental and ecological impacts, even in countries far away from pollutant-emitting source areas in other countries.

### The Swedish case study and the OECD project

Two years after Odén’s article, the Swedish government decided to prepare a “case study” as a contribution to the UN Conference on the Human–Environment in Stockholm 1972 (Royal Ministry of Foreign Affairs and Royal Ministry of Agriculture 1972). Bert Bolin at the Stockholm University was appointed chair of the study, which included Svante Odén, Henning Rodhe, and Lennart Granat as authors. The report included a broad environmental assessment of the sulphur emission problem including sources, atmospheric and surface water chemistry, and effects on ecosystems and materials. Finally, it also included scenarios and estimated costs for environmental damage and control; in fact it was probably the first full systems analysis of an environmental problem.

In the report, a first estimate was made of the relative contributions of domestic and foreign emissions to the sulphur deposition in Sweden (Rodhe 1972). Estimates were also made of the effects of sulphur emissions on excess mortality and showed that 50% of the Swedish lakes and rivers would reach a critical pH level within 50 years (assuming continuation of present emission trends). Even if some aspects of the report received criticism, the overall case study was well received by the UN conference and in its final report (see http://www.un-documents.net/aconf48-14r1.pdf) regional air pollution was explicitly mentioned (§85) with a citation of the Swedish study.

The Swedish initiative in the OECD resulted in a collaborative project to investigate the nature and magnitude of the transboundary transport of emitted sulphur dioxide over Western Europe, in which 11 countries participated. To initiate the project, a Nordic organisation on scientific research, Nordforsk, was asked to plan and develop methodologies for the investigation. Scientists and institutions from Norway, Sweden, Denmark, and Finland established an expert group in April 1970, which became central for the development and implementation of the OECD project. The Norwegian Institute for Air Research (NILU) offered through its director Brynulf Ottar to coordinate the project. The project included emission inventories, measurements of atmospheric concentrations, and deposition, together with model development and application for the assessment of the transport. A key part of the model calculations was to prepare the so-called “blame matrices”, through which the transport of pollutants between countries could be quantified.

The main conclusion from the OECD project, published in 1977, was that “Sulphur compounds do travel long distances in the atmosphere and the air quality in any European country is measurably affected by emissions from other European countries” (OECD 1977). Even if there still were hesitations about the magnitude of the transport, the common opinion was that transboundary transport of air pollution is an issue that needs collaboration across national borders. These conclusions paved the road for a pan-European scientific collaboration on air pollution, the European Monitoring and Evaluation Programme (EMEP) starting in 1977. The findings from the project also formed the basis for the Air Convention (Table 1). EMEP was already from the beginning included in the Convention as a key element, strongly contributing to the scientific credibility of the policy work.

### Threats to forests boosted the interest

In 1980, the German scientist Bernhard Ulrich warned that European forests were seriously threatened from atmospheric deposition of sulphur. From his long-term experiments in the Solling area, he concluded that the high deposition of atmospheric pollutants had seriously changed the soil chemistry (Ulrich et al. 1980). Ulrich pointed to the links between sulphur deposition and the release of inorganic aluminium. His findings became a policy issue not only in Germany but in Europe as a whole, and even in North America. The alarms—often exaggerated—went like a wildfire through media and changed many attitudes throughout Europe. Newspapers were filled with photos of dying forests, in particular from “The Black Triangle”, the border areas between Poland, East Germany, and Czechoslovakia, characterised by large combustion of brown coal with high sulphur content. Forest inventories showed crown thinning and other effects on forests, but it became difficult to finally determine that acid deposition was the (only) cause for the observed effects.

The increasing interest in regional air pollution also paved the way for the first international agreement on emission control under the Air Convention. As a start, countries with a large interest in taking actions formed a “club” under the Convention, aiming for a 30% reduction in emissions. This ambition then became the basis for the first emission reduction protocol, the Sulphur Protocol signed in 1985. While Germany and some other West European countries acted almost immediately on the alarms, the progress in emission control in Eastern Europe was very slow during the 1980s, even though several of these countries signed the protocol. In fact, substantial decrease in emissions did not take place in the East until after the break-down of the communist regimes and the industrial collapse around 1990.

### Critical loads and advanced policies

**Fig. 4:**
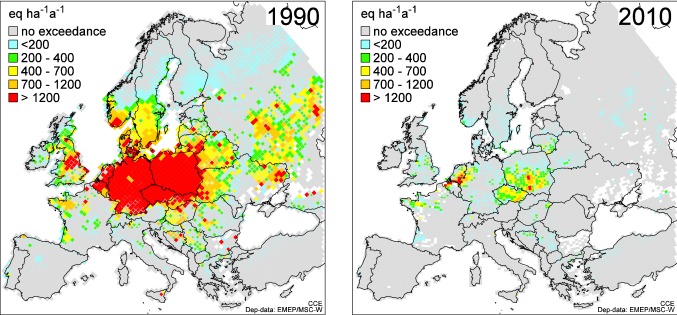
The outcome of emission control of SO2, NOx, and NH3 between 1990 and 2010 presented as maps on exceedance of critical loads of acidity. Such maps have played an important role for illustrating outcomes of future policies as well as of actions taken (from Maas and Grennfelt 2016)

One of the most well-known characteristics for the control of the acid rain problem is the concept of Critical Loads (Nilsson 1986; Nilsson and Grennfelt 1988). The Executive Body, the highest decision-making body of the Air Convention, decided in 1988 that new negotiations on the control of sulphur and nitrogen emissions should be based on critical loads, and all parties to the Convention were requested to prepare their own critical load maps. The Netherlands offered to take a lead and prepared mapping manuals and initiated an international network, which became crucial for the scientific and policy acceptance of the concept (Hettelingh et al. 1991; De Vries et al. 2015; Fig. 4). (The critical loads concept is further discussed later in the paper)

When critical loads became a basis for further protocols, Integrated Assessment Models (IAMs) offered a method to calculate how to achieve a prescribed ecosystem effect reduction in the most cost-effective way. A couple of different approaches were developed, but the model at the International Institute for Applied Systems Analysis (IIASA) became the official model on which the Second Sulphur Protocol signed in 1994 was agreed (Hordijk 1995).

When revising or developing a new protocol for nitrogen oxides the concept could, however, not be used in the same way as for sulphur and acid deposition, since the NO_x_ emissions contributed to several effects and, in addition, a strategy would need to take additional compounds into account. Instead, a more advanced approach was suggested by which both several effects and several compounds could be considered simultaneously (Grennfelt et al. 1994, Fig. 5). IIASA and other bodies under the Air Convention were asked to develop an integrated assessment model that fitted into a broader approach and a more comprehensive model was developed, which made it possible to simultaneously take into account the effects of acidic deposition, nitrogen deposition, and ozone—the so-called multi-pollutant, multi-effect approach. The calculations became the basis for the Gothenburg Protocol (GP) that was signed in 1999 (Amann et al. 1999). The GP and the parallel EU National Emissions Ceilings (NEC) Directive from 2001 outlined control measures for 2010 and beyond.

**Fig. 5:**
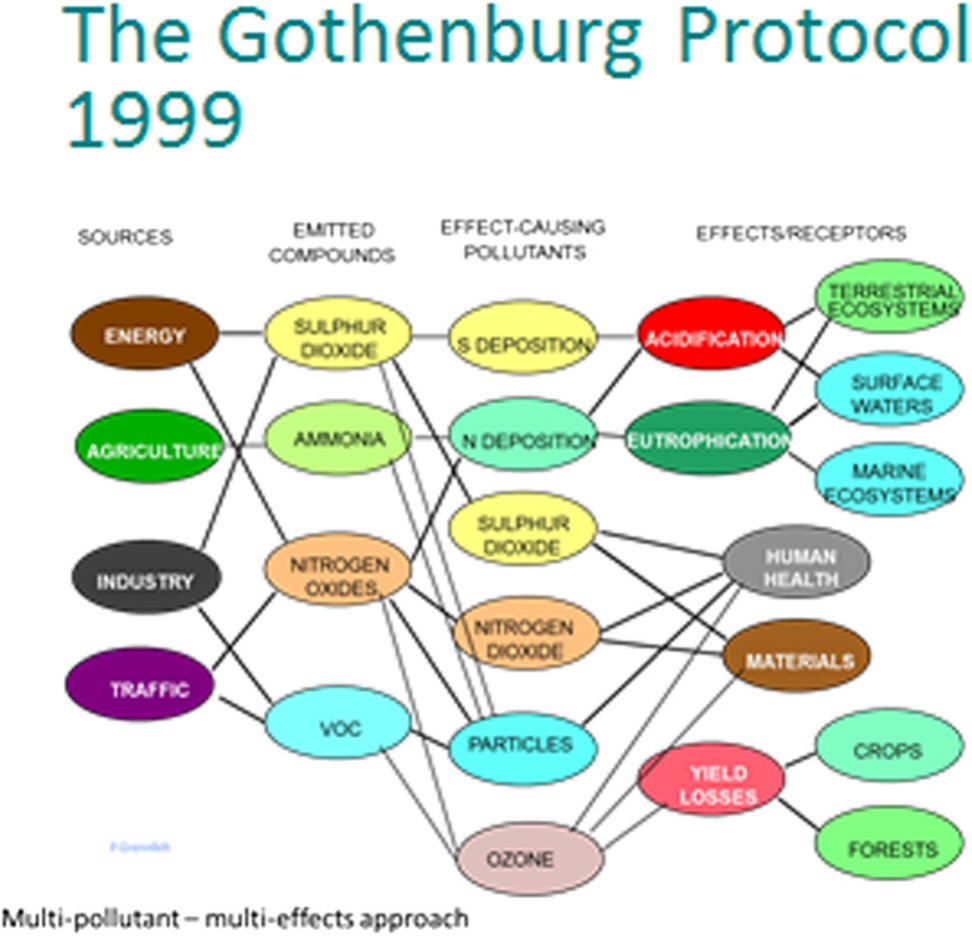
Links between sources and effects used as an illustration in the preparation of the Gothenburg Protocol. From Grennfelt et al. 1994

### After 2000—Health effects and integration with other policies became main drivers

The basis for the GP was almost entirely ecosystem effects. Around 2000, however, public health effects from air pollution became increasingly important. Large epidemiological studies indicated that air pollution was a significant source of premature deaths and that particles were a main cause of the health effects (WHO 2018). When the European Commission started its work to revise the NEC directive, health effects became central and the Air Convention followed. Further studies have supported the role of air pollution for health effects and when the GP was finally revised in 2012, health effects dominated as a policy driver for the establishment of national emission ceilings, and for the first time particulate matter was included in an international protocol (Reis et al. 2012).

When considering further actions after signing the GP in 1999, it was realised that for some pollutants under the Air Convention, emission control needed to be considered over larger geographic scales than Europe and North America alone. Ozone was of particular importance, since long-term objectives in the form of critical levels and public health standards could not be reached without taking into account sources outside the areas considered so far. Future policies therefore needed to include the ozone precursors methane and to some extent carbon monoxide. A task force on Hemispheric Transport of Air Pollution (HTAP) was set up under the Convention in 2004, with a primary objective to quantify the intercontinental transport of pollutants. The outcome of its work clearly showed the importance of considering air pollution in a wider geographic perspective than had been done so far (Dentener et al. 2010).

Climate change has for more than a decade become an issue of increasing interest for air pollution science and policy. In many cases, the emission sources are the same and there are obvious co-benefits (and some trade-offs) in handling them together. One aspect that has received large interest is the option to decrease short-term temperature increase through control measures directed towards atmospheric pollutants that also contribute to the warming of the atmosphere, in particular black carbon and methane (for methane both by itself but also as a tropospheric ozone precursor) (Ramanathan et al. 2001). Compounds contributing to both air pollution effects and to the radiation balance in the atmosphere have been named Short Lived Climate Pollutants (SLCPs). SLCPs thus also include compounds that are cooling the atmosphere, i.e. small secondary aerosols, e.g. sulphate particles. Recent research has focused on a better understanding of these compounds’ contribution to both air pollution and climate as well as on opportunities for selective control of these compounds (e.g. Sand et al. 2016).

Reactive nitrogen species are another group of compounds that has received increased attention after the turn of the century. Around 2006 several initiatives were taken in Europe, including a special task force on Reactive Nitrogen under the Air Convention, a large-scale EU project on nitrogen, and the preparation of a European Nitrogen Assessment (Sutton et al. 2011). Here nitrogen was considered both as a traditional atmospheric pollutant and within a societal and industrial context. A cascade perspective, where one fixed nitrogen molecule could contribute to a series of effects before it returns to molecular nitrogen again, was introduced (Galloway et al. 2003). The studies have pointed to the importance of the agricultural sector for the intensification of reactive nitrogen cycling, determined by food production mechanisms and dietary choices.

### North America

In North America, the acid rain problem developed to a large extent in parallel with the situation in Europe. Lake acidification became already from the beginning a main driver, and monitoring programmes were set up both in the United States and Canada (Driscoll et al. 2010). The US National Atmospheric Deposition programme (NADP) started in 1976 and is still running. Both countries have taken part in the Air Convention activities and have signed most of the protocols and achieved decreases in SO_2_ emissions of the order of 80% between 1980 and 2015. The US has however taken a different approach with respect to policy in comparison to Europe. Instead of developing a strategy based on integrated assessment modelling, it was decided to establish an emissions trading programme for the large electric generation sources under the Clean Air Act (See also UNECE 2016).

## Characteristics of the science–policy interactions

In this section we will, from a science–policy perspective, briefly discuss some characteristics of the history of acid rain and transboundary air pollution that have become central for the international collaboration, not only on air pollution but also for international environmental collaboration in general. We will bring up monitoring, modelling, and data collection (including field experiments and long-term studies carried out in order to understand and quantify effects to ecosystems), development of bridging concepts that have served the implementation of strategies, and finally the dynamics in the science–policy interactions.

### Monitoring, modelling, and data collection

**Fig. 6:**
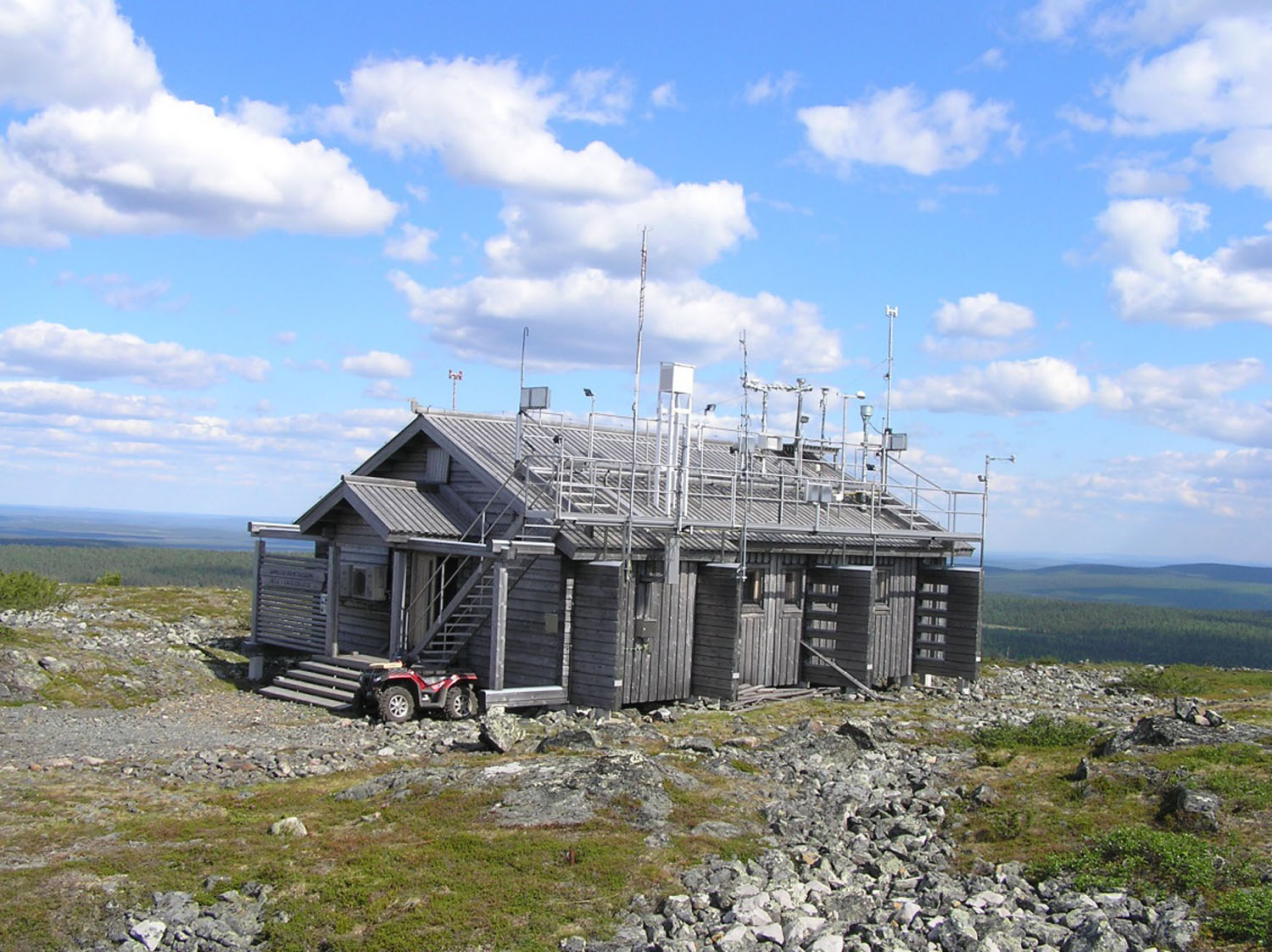
Atmospheric monitoring stations have been of importance for understanding the long-range transport and chemical conversions of atmospheric pollutants. Pallas air pollution background station in Northern Finland (Photo Martin Forsius)

Monitoring of atmospheric concentrations, deposition, and ecosystem effects has been a key for understanding the causes, impact, and trends in acid rain, both in Europe and North America and later in other geographic areas (Table 2). The original EMEP network has since the start over 40 years ago formed a broad atmospheric monitoring system. The originally established simple monitoring stations have over time been complemented with more advanced monitoring, and some stations are today advanced atmospheric chemistry platforms with continuous collection of a multitude of atmospheric parameters (Fig. 6). The EMEP database is nowadays widely used for a variety of scientific purposes including computation of long-term trends, exposure estimates, and as a basis for modelling. EMEP has also become a model for monitoring networks related to other geographical regions, conventions, and purposes. One example is the acid deposition monitoring network in East Asia (EANET). It is obvious that having a qualified centre for data collection and storage, standardisation, and intercalibration of methods has served the international policy system extremely well. Its open nature is part of the success. The financial support to EMEP, regulated through a separate protocol, has been fundamental for the development and progress of the monitoring activities.

**Table 2:** Long-term monitoring activities in relation to acid rain and other pollutants

Activity and time	Geographical coverage and number of sites	Programme centre	Web page comments
*Atmosphere*			
EACN (IMI network) 1955–1976	Europe > 100 sites	Stockholm University	Some sites continued within EMEP after 1976 L Granat, pers. comm.
WMO GAW/BAPMoN 1964–	Global > 200 sites	World Meteorological Organisation	http://www.wmo.int/pages/prog/arep/gaw/gaw_home_en.html
EMEP 1977–	Europe and ECE region of Asia approx. 350	Norwegian Institute for Air Research (NILU)	http://www.emep.int/
NADP 1977–	US approx. 260 sites	University of Wisconsin-Madison	http://nadp.slh.wisc.edu/
CAPMoN (incl. APN) 1978–	Canada 25–35 sites	Environment Canada	https://www.canada.ca/en/environment-climate-change/services/air-pollution/monitoring-networks-data/canadian-air-precipitation.html
EANET	East Asia	Asia Center for Air Pollution Research (ACAP)	http://www.eanet.asia/
Male Declaration 2003–	South Asia 15 sites	Asian Institute of Technology	http://www.rrcap.ait.asia/male
*Ecosystems*			
ICP Forests 1985–	Europe 5000 sites and 500 intense sites	Thünen Institute of Forest Ecosystems	http://icp-forests.net/
ICP Waters 1985–	Europe and North America approx. 250 sites	Norwegian Institute for Water Research (NIVA)	http://www.icp-waters.no/
ICP Materials 1985–	Europe and North America approx. 40 sites	Rise KIMAB AB, Sweden	http://www.corr-institute.se/icp-materials/web/page.aspx
ICP Integrated Monitoring 1987–	Europe approx. 50 sites	Finnish Environment Institute (SYKE)	http://www.syke.fi/nature/icpim
ICP Vegetation 1987–	Europe	Centre for Ecology & Hydrology, UK	https://icpvegetation.ceh.ac.uk/

Monitoring of air pollution effects in a systematic way under the Air Convention started a few years later than EMEP and was organised through so-called International Cooperative Programmes (ICPs). Separate programmes were set up for forests, waters, vegetation (primarily ozone), materials, and integrated monitoring. A separate ICP was set up for developing critical load methodologies and coordinating European-scale mapping activities (ICP Modelling and Mapping). The ICPs are of great importance for general understanding of the magnitude and geographical distribution of the effects and for showing how decreases in emissions have led to beneficial conditions in ecosystems and decreased material corrosion (Maas and Grennfelt 2016). Ecosystem monitoring is also important for the development and verification of ecosystem models. Since their start, the responsibility for the ICPs has been taken by different parties of the Air Convention (Table 2). The distributed responsibility has been of large importance for the establishment of networks of monitoring sites among the Convention parties, but the system has not had a stable financial support in the same way as for EMEP. This has resulted in the lack of a common source for easily accessible data or adequate resources for standardisation and intercalibration.

Monitoring and other data collection (i.e. emissions and critical loads) under the Air Convention are responsibilities of every country, and data are then used for the assessments on the Convention level as well as for the development of EU air pollution policies. The bottom-up process in data collection is important for the development of national expertise and, not the least, for the establishment of national policies. In this way, direct communication links between the science and the policy levels within countries have evolved.

Numerical modelling of atmospheric pollution is also a long-term commitment under EMEP. The atmospheric chemistry models are necessary for the understanding of the nature of transboundary transport but also to make budget estimates of the exchange of pollutants over Europe and North America, and later on a hemispheric scale. The Meteorological Synthesizing Centre West at the Norwegian Meteorological Institute together with the Eastern Centre in Moscow took the lead in this work. In addition to calculating transboundary fluxes, the centres are important for coordinating modelling efforts done by other groups, forming a basis for scrutinising models and support further modelling.

### Field experiments and long-term studies—a way to understand processes and trends, and to visualise the problems

**Fig. 7:**
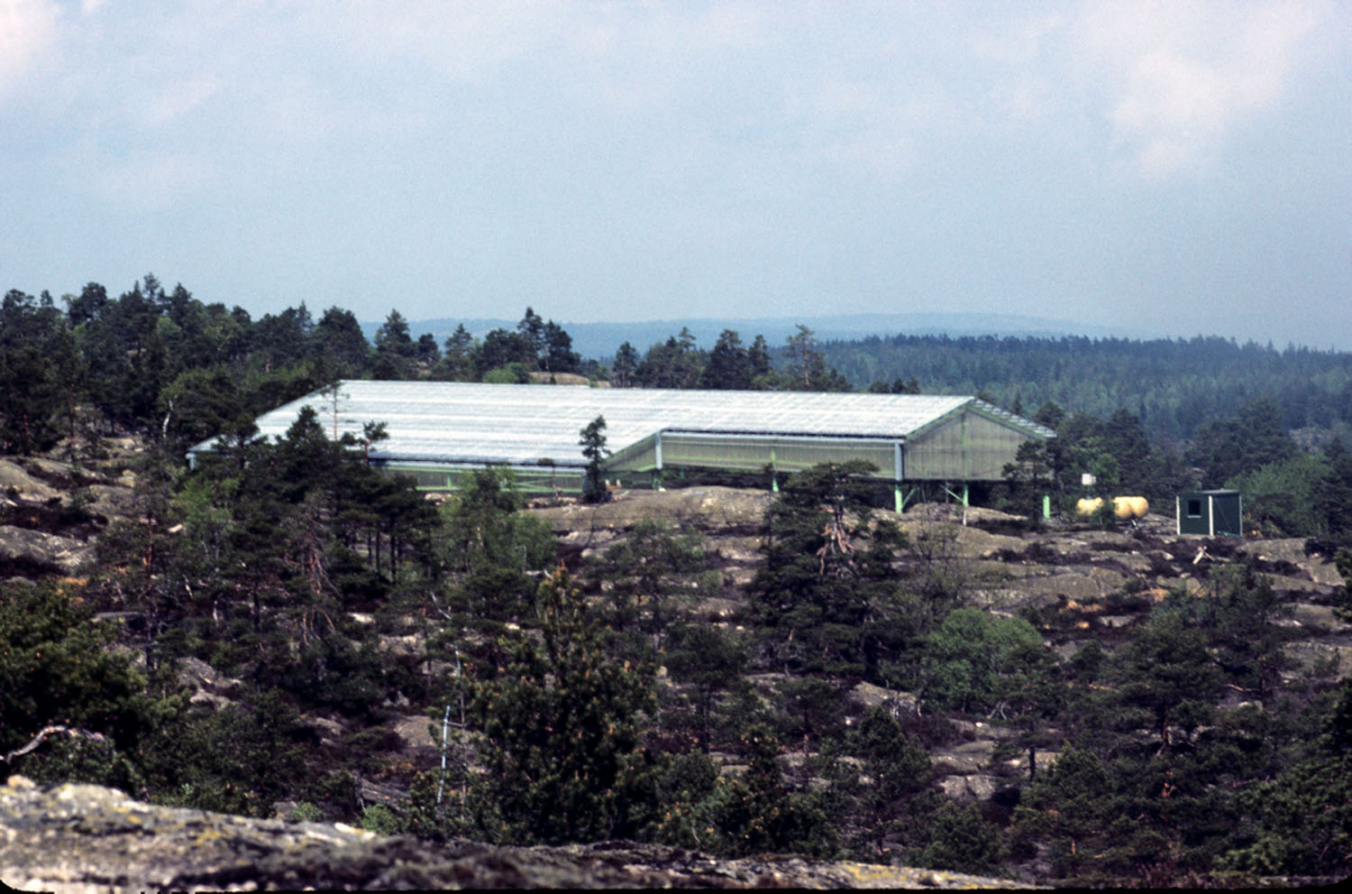
Field experiments have played an important role for the overall understanding of the interactions between atmospheric deposition and ecosystem effects. The photo illustrates the covered catchment experiment to study the recovery of ecosystems at reduced emissions in Risdalsheia Norway (Photo NIVA)

Some of the most important and reliable findings regarding acid rain and its effects on ecosystems emanate from long-term field experiments. These experiments, which are known from the sites where they are run, include Hubbard Brook (US), Solling (Germany), Risdalsheia (Norway) and Lake Gårdsjön (Sweden) (Fig. 7). The studies there have shown how acid deposition and the impact of other air pollutants have changed the ecosystems, but also how ecosystems respond to decreased emissions (e.g. Wright et al. 1988; Likens et al. 1996). A central feature in all these field experiments was the establishment of ion budgets, from which the chemical effects on acid deposition can be analysed and understood (Reuss et al. 1987).

In the intense research period during the 1970s and 1980s, a number of large-scale research programmes and experiments of temporary nature were set up, some of them in connection with the above-mentioned sites. The first research programme of some magnitude was the Norwegian programme “Acid precipitation—effects on forest and fish” (SNSF), which run between 1972 and 1980 (Overrein et al. 1981). At that time the scientific understanding was limited, and the programme received a lot of attention. The results were important for the general acceptance that long-distance transport of sulphur caused acidification of surface waters, with a serious die-off of fresh water fish populations (salmon and trout) as a main consequence. On the other hand, the studies on Norwegian forests did not give any significant evidence for acid rain effects. The SNSF project was a joint effort across disciplinary and organisational boundaries, with scientists mainly from the research institute sectors outside of traditional academia. This project served as a model for later research programmes and provided educational opportunities for a new generation of scientists working together on all aspects of the acid rain issue—emissions and their control, atmospheric transport and deposition, impact on ecosystems, health and materials, and finally development of pollutant-control policies.

The long-term field experiments served another important task. The sites became exhibition platforms, at which policymakers, experts, scientific journalists, and leaders of non-governmental organisations (NGOs) and others can be informed about the problem directly on site. During the most intense period in the 1980s and early 1990s, politicians and industry leaders, often directly involved in decisions on the highest levels, visited many of these experimental sites. For example, US congress members travelled across Europe to see and understand the issue in preparation for the 1990 amendment of the Clean Air Act.

### Bridging concepts and approaches

Concepts developed, such as critical loads and similar approaches, formed links between science and policy, and were essential for the understanding and scientific legitimacy of the policy measures. These concepts also formed a basis for priority setting in agreements under the Convention and the EU, but also to some extent for national policies. Even “acid rain” can be considered as a bridging concept. While the acidity from sulphur and nitrogen compounds is threatening ecosystems through a chemical change, the expression also gives the impression of a threat to the life-giving rain, a fundamental necessity for life on Earth.

The quantification of transboundary fluxes was very important politically. The establishment of national budgets and so-called blame matrices formed the first bridging concept. The development of mathematical models to calculate source–receptor relations was a scientific challenge but when the annual tables were prepared showing the interdependence between countries with respect to atmospheric emissions and deposition, they served as an important basis for the need for common action. Anton Eliassen, the leader of the modelling centre at the EMEP Meteorological Synthesising Centre West (MSC-W) during many years (the Eastern center is in Moscow—MSC-E), was key to this development as well as for the communication of the results to policymakers.

As earlier mentioned, critical loads played an outstanding role for the development of the more advanced strategies leading to the Second Sulphur Protocol and the GP. Critical loads formed a successful link between science and policy that became crucial for the negotiations and agreements. The concept, first discussed in 1982, was taken from the original idea to application quite quickly during the 1980s. The Swedish expert Jan Nilsson was a key leader for the success of the concept, and the Nordic Council of Ministers played a unique role for forming the links between science and policy. Through a series of workshops involving both key scientists and key policymakers, the concept gained the legitimacy on which policies were developed. According to Jan Nilsson, it all started with requests from both industry and negotiators to have a sounder base for emission control, something that could express the long-term objectives for emission control policies. The concept was first met by scepticism, not least from scientists, but after a couple of workshops, the interest turned around and the concept became widely accepted (Nilsson 1986; Nilsson and Grennfelt 1988). When critical loads were included in the plans for the next rounds of the sulphur and nitrogen protocols in 1988, it changed the way the Air Convention operated.

The application of the critical loads concept has encouraged intense research over several decades where the main objective has been to find simple chemical parameters that can mimic the (often biological) real effects or effect risks. For lake acidification, where the effects of dissolved aluminium on fish often were chosen as the main biological effect, the acidity of the water, mostly expressed as acid neutralising capacity (ANC), is used (e.g. Henriksen et al. 1989; Forsius et al. 2003; Posch et al. 2012). For forests, where the toxicity of aluminium to tree roots is considered as critical, the Al^3+^ to Ca^2+^ ratio in soil water has become the main effect parameter (Sverdrup et al. 1990; de Vries et al. 1994).

Integrated assessment modelling (IAM) also has been a bridging concept. The idea of applying systems analysis goes back to the work at IIASA in the beginning of 1980s. A conceptual model was formulated by Joseph Alcamo, Pekka Kauppi, and Maximilian Posch for the interactions between emissions, their control (including costs), and the effects on ecosystems (Alcamo et al. 1984). Their work of bringing together the scientific knowledge to a comprehensive systems analysis tool formed a new way of framing environmental policies. Under the leadership of Leen Hordijk, the new idea was introduced to and accepted by the policy side, which had asked for more targeted methods for policies than simple percentage decreases in amounts of emissions. IAMs as a policy-supporting concept was then taken further by Markus Amann, who led the development of the more advanced RAINS (later GAINS) models that were used as a basis for the GP and later agreements (Amann et al. 2011). From the strategies strictly directed at ecosystem effects, the approach is now widened to include health effects, local air pollution impact, climate policies, and reactive nitrogen.

**Fig. 8:**
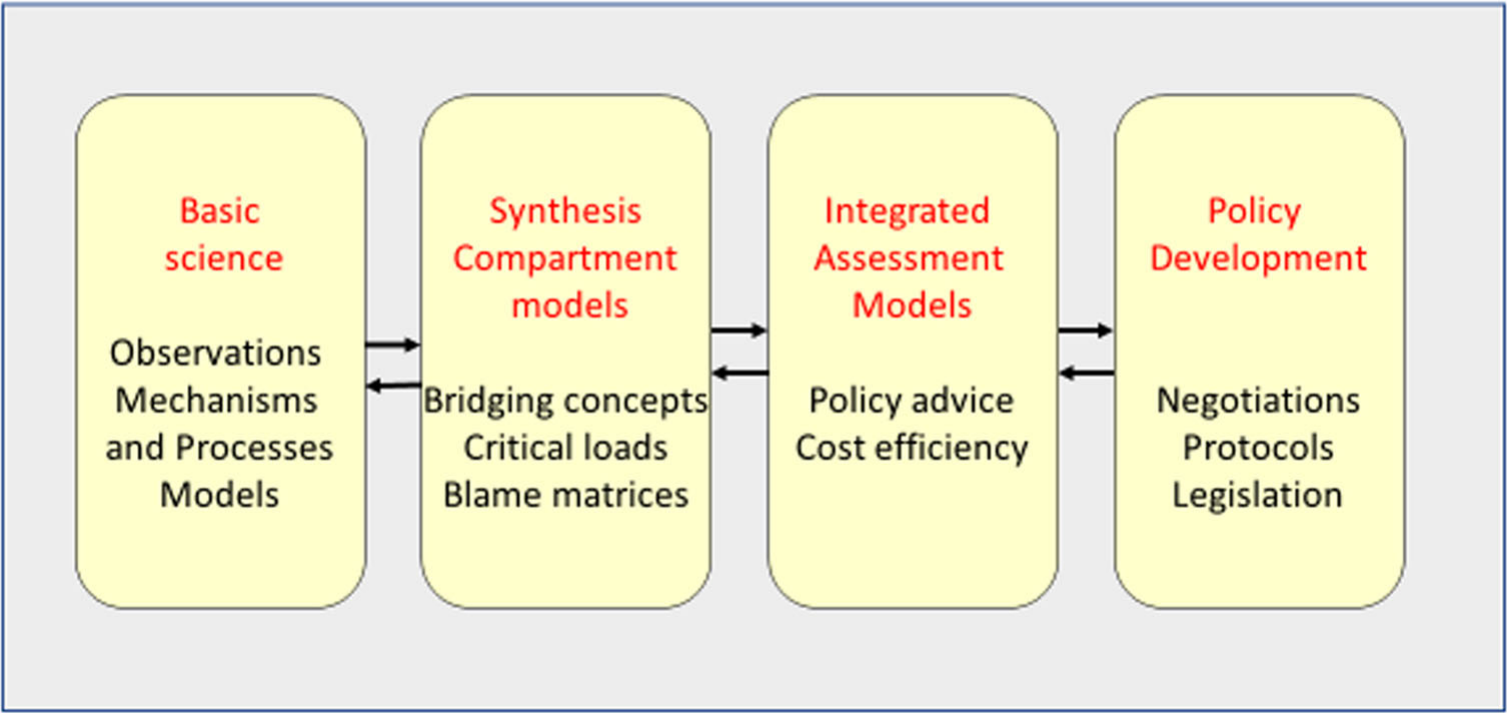
The scientific support to regional air pollution policies consists today of a series of steps. The policy side may often only see the integrated assessment step and not realise that the legitimacy of the use of scientific support builds on an advanced system of underlying research and development

All the bridging concepts are to varying degrees dependent on underlying models, assumptions, and simplifications. For these to be accepted among policymakers, it is important to keep transparency and confidence in the underlying data and to scientifically evaluate and scrutinise them. This is particularly important for the IAMs, which are the final step in a chain of inputs (Fig. 8). The models have often been criticised, not least from industry and other stakeholders that are questioning the priorities that result from the IAM calculations. IIASA, as a provider of the model calculations, has, however, been transparent, and countries and stakeholders have always had the option to re-check data and take this into account when developing their own negotiation positions.

### Forming science–policy credibility

In all interactions between science and policy, it becomes crucially important to maintain scientific credibility. The close involvement of scientists has been a signature of the Air Convention. Scientists have always had a role at the policy meetings, communicating results from basic scientific research over outcomes of monitoring and inventories to presenting options for control strategies. Scientists have in this way taken the responsibility to move scientific knowledge into the policy system and presenting results in a way that has been understandable and useful for the policy work. The role of the scientists has been as *honest brokers*, not that of *issue advocates* to follow the terminology of Pielke (2007). The leadership from the policy side and its sensitivity to changes in the underlying science and observations of new problems have also been important, and have resulted in repeated changes in the framings of the Air Convention to adapt to new situations: going from an initial framing around sulphur and acidification, through extension to eutrophication, human health, materials, crops, biological diversity, and finally to links to climate, urban air quality, and societal changes. A balanced interplay between the two communities has in this way been developed and maintained over time.

Another factor is the building of networks. The strong networks of scientists and policymakers pushed the politicians. The whole field of international diplomacy during these four decades of the Convention is built on incremental developments forming protocols of increasing capability to solve specific environmental issues by cutting emissions in a cost-effective way.

## Future challenges

### New approaches necessary

International air pollution control is by many considered as a success story. However, the success is in many ways limited to Europe and North America and a few additional industrialised countries (including Japan and Australia), where emissions of sulphur dioxide, nitrogen oxides, VOCs, and some other compounds have been decreased significantly (Maas and Grennfelt 2016). But even in the areas, where air pollution has been a top priority for several decades, air pollution remains a problem. Ecosystem effects, which were the main reason for the establishment of the Convention, are to some extent reduced, but the acidification effects of historical emissions will remain for decades (Wright et al. 2005; Johnson et al. 2018) and the emissions of ammonia have so far only been reduced by 20–30% in Europe and even less in North America. Looking at health effects, it is difficult to talk about success, when hundreds of thousands of inhabitants on both continents are predicted to meet an earlier death due to air pollution.

But the problem is even larger and more urgent when looking outside the traditional industrialised world. The focus is today on the large urban regions in the countries that are facing rapid population growth and industrialisation. Although large efforts now are being made to decrease sulphur emissions in China—the world’s leading sulphur emitter—major challenges remain. In India and several other countries, sulphur emissions are still increasing. Estimates indicate that more than four million people die prematurely due to outdoor air pollution globally (https://www.who.int/airpollution/ambient/health-impacts/en/). It is assumed that fine particles (PM2.5) are a main cause for the health effects. The new and great challenge is therefore to control air pollution in relation to health risks, in particular by decreasing exposure to the small particles.

There is, however, a risk that control measures will only to a limited extent focus on the right sources and the right measures. In Paris, several air pollution episodes with high concentrations of particles have occurred during recent years. At first, these episodes were considered to be caused essentially by local emissions. More thorough analysis has, however, shown that they were to a large extent caused by regional emissions and buildup of high concentrations over several days when urban emissions of oxides of nitrogen from traffic mix with ammonium emissions from surrounding agricultural areas to form particulate nitrate. Similar situations are also often encountered in urban regions in developing countries, e.g. by agricultural waste burning, and need to be considered. Air pollution problems are, as previously mentioned, also linked to intercontinental and hemispheric scales.

It is also obvious that the research communities within air pollution and climate change need to work more closely together. Health aspects are of importance both from air pollution and climate change perspectives, and heat waves carry poor air quality as winds are often very low and the atmospheric boundary layer stagnant. During heat waves, the soil and vegetation dry up and increase the likelihood of fires, which also can cause severe air pollution, as seen in wildfires around the world (e.g. California in 2018).

Despite the large progress in atmospheric and air pollution science, basic questions still need further investigations to develop the best policies. Such areas include a better understanding of health effects from air pollution, nitrogen effects to ecosystems, and air pollution interactions with climate through carbon storage in ecosystems and impacts on radiation balances. Modelling is a scientific area where much progress has been made and where increased computer power, as in climate change research, has allowed integration of atmospheric chemistry into the climate models formulated as Earth system models, coupling the atmosphere, ocean, the land surface, cryosphere, biogeochemical cycles, and human activities together. This has allowed studying air pollution and climate change simultaneously. The modelling approach can be further developed when observations are designed to map Earth system component boundaries to understand and quantify the flows and interactions between different compartments, including terrestrial and aquatic ecosystems. Air pollution should be an integrated part of such models. In this context, global-scale concepts such as “planetary boundaries” and “trajectories of the Earth system vs. planetary thresholds” have been developed (Rockström et al. 2009; Steffen et al. 2018).

### Solutions are available; driving forces and investments are lacking

In 2016, the Air Convention launched a scientific report “Towards Cleaner Air”, in which the actual air pollution situation within the UNECE region was updated (Maas and Grennfelt 2016). The report also presented future challenges and ways forward to solve the air pollution problems. It also showed that solutions are available for most of the identified problems at affordable costs below the health and ecosystem benefits of the control actions.

Even if solutions are available, many parts of the world are facing large problems in implementing them. There are several reasons, but often there is a lack of knowledge and resources. This is particularly true in many developing countries. Another reason is the lack of political interest. Air pollution is still not of top priority among politicians, even if there is overwhelming evidence that air pollution is one of the most common causes of shortened life expectancies. Another reason may be that other interests (e.g., industry and agriculture) are forming strong lobbying forces delaying actions.

Air pollution is a problem that cannot be seen in isolation. Future policies need to take into account climate change and climate change policies. Whereas some air pollutants—in particular black carbon particles—contribute to warming, others, including sulphate particles, tend to cool the climate. A reduction in sulphur dioxide emissions, although highly desirable from health and ecosystems perspectives, will therefore contribute to warming. On the other hand, a reduction of black carbon will be a win–win solution. It is also important to see air pollution control in the perspective of sector policies, such as energy, agriculture, transportation, and urban planning in order to meet the challenges to decrease air pollution problems.

### Internationally coordinated actions and infrastructures are keys for success

The perspective of international cooperation on air pollution is changing. Policy development is no longer limited to long-range transport in line with that developed under the Air Convention. The ranking of air pollution as a top ten cause of premature deaths in the world has given high priority to the issue within fora such as the WHO and UN Environment. Both organisations have adopted resolutions calling for actions (WHO 2015; UN Environment 2017). Additional initiatives are taken by other organisations, such as the World Meteorological Organisation (WMO), the Climate and Clean Air Coalition (CCAC), and the Arctic Monitoring and Assessment Programme (AMAP). WMO is particularly important as a global technical agency for weather and climate observations, research and services, and it is rapidly developing its regional and global capacities in Earth system observations, modelling, and predictions to the benefit of mitigating a range of environmental threats and for global use. The research is done in large programmes like Global Atmosphere Watch (GAW) and the World Weather Research Programme (WWRP). Even if the starting point and modes of action can be different, all initiatives are aiming for the same goal, cleaner air. It is also worth mentioning the initiative taken by the International Law Commission, under which a proposal for a Law for the Protection of the Atmosphere has been prepared (http://legal.un.org/ilc/summaries/8_8.shtml) but in the current international atmosphere there is a lack of political support to implement it. Our hope is that the situation will change soon—the initiative is too important to fail.

The UN has put forward a very strong agenda in order to reach the Sustainable Development Goals (SDGs), and air pollution is an integral part of several of the SGDs, like goal No 2: No Hunger, No 3: Good health and well-being, No 6: Clean Water, No 7: Affordable and clean energy, No 9: Industry, innovation and infrastructure, No 11: Sustainable cities and communities, No 13: Climate action, No 14: Life below water, No 15: Life on Land, No 16: Peace and Justice, and No 17: Partnerships for the Goals. The approach taken to develop multiple pollutant—multiple impacts protocols under the Air Convention can serve as important learning ground to meet the ambitions of many of the SDGs. Air pollution plays an integral role in the evolution of the food production and ecosystem services, the health of the population, the shape of the energy and transportation systems, and the availability of clean water. Climate change is a very significant common and cross-cutting factor.

The Air Convention has taken some steps in promoting air pollution on a wider scale. Due to its long history and well-developed structure, it has taken a role of making sure that international organisations having air pollution on its agenda are aware of each other and to invite to further collaboration and development. Initiatives are taken both within the formal Convention structure and through dedicated workshops (UNECE 2018; Engleryd and Grennfelt 2018). The approach developed under the Air Convention, which has proven successful in linking scientific evidence, monitoring, and integrated assessment modelling directed towards cost-effective solutions, may also serve as a working model for environmental problems in other fields.

These new international initiatives have a strong emphasis on policy development. The experience from the 50 years of international air pollution development is the value of well-defined scientific objectives and activities supporting policy. The increased interest from WHO and UN Environment is welcome and there are expectations of an active role from these organisations in combatting the situation in many parts of the world. However, for these organisations, air pollution is just one of several priority areas, and priorities may change. Further, none of these organisations are likely able to set up advanced infrastructures with respect to emission inventories, monitoring, and research. Here WMO needs to live up to its mission and capitalise on global research and development efforts and improve the global operational capability to observe, analyse, and forecast the development of the Earth system and its components, air pollution being an important part. This is in line with the WMO strategic plan and with fast growing capabilities in some countries and in global centres like The European Centre for Medium Range Weather Forecast (ECMWF). WMO, through GAW, is also developing a research-driven operational system (IG3IS) for top-down determination of greenhouse gas emissions, to complement the usual bottom-up-based inventories where emission factors and fuel consumption or production statistics form the basis for the emission estimates (https://library.wmo.int/doc_num.php?explnum_id=4981). The Air Convention and the science support for the policy work there has been a model for the WMO ambitions on a global basis. However, current investments in these new capabilities are not enough to get the societal return they would offer.

Therefore, we see a need for developing long-lasting infrastructures that can continuously develop science-based control policy options, potentially as part of a wider network of global observatories for comprehensive monitoring of interactions between the planet’s surface and atmosphere (Kulmala 2018). Such a network should be able to support policies from local to the global levels. The challenge is how to organise and raise resources for scientific support on a wider scale. Financial institutions such as the World Bank and/or regional banks may step in and make sure that control measures and investments are made on a sound basis with respect to global air pollution.

There is also a need to mobilise new generations of scientists, scientists that are willing to cross boundaries and focus on thematic problems and to build legitimacy among policymakers (e.g. Bouma 2016). Today we have more developed and stronger political institutions to handle environmental problems, which may make it harder for scientists and individuals to influence and make a difference. It is also important to mobilise new generations of dedicated policymakers. Unfortunately, we also see that politicians often are questioning science and seeing science as just a special interest. Public awareness may be a key for forming stronger interests and put pressure on decision-makers. During the acid rain history, NGOs played an important role in driving the awareness at a wider scale than local or national actions and could be important for a more global movement towards cleaner air. We also see the need for a deeper responsibility not only from politicians but also from industry. The so-called “diesel gate” exposed the cynic view from parts of the industry to peoples’ health, which hopefully will not occur in the future. Instead we hope that it was an eye-opener and that industry instead can play a role as a forerunner and a positive power for a cleaner atmosphere.

## Final remarks

The Acid Rain history taught us that when science, policy, industry, and the public worked together, the basis was formed for the successful control of, what was considered, one of the largest environmental problems towards the end of the last century. We learnt from experience that science-based policy advice worked well when the best available knowledge was provided, and used to understand the specific problems, generate, and evaluate the policy options and monitor the outcomes of policy implementation.

However, the world does not look the same today, and we cannot just apply the ways the international science community worked together then on today’s problems. But there are lessons to be learnt. Most important is the building of mutual trust between science advisers and policymakers, and that both communities are honest about their values and goals. In this way, a fruitful discussion around critical topics within society can be formed. *The advice works best when it is guided by the ideal of co*-*creation of knowledge and policy options between scientists and policymakers* (SAPEA 2019).
